# Functionalized Triblock Copolymers with Tapered Design for Anion Exchange Membrane Fuel Cells

**DOI:** 10.3390/polym16162382

**Published:** 2024-08-22

**Authors:** Ming-Tsung Lee

**Affiliations:** Department of Chemical Engineering and Biotechnology, National Taipei University of Technology, Taipei 10608, Taiwan; mtlee@ntut.edu.tw

**Keywords:** tapered block copolymer, anion exchange membrane, fuel cell, dissipative particle dynamics, ion conductivity, SEBS

## Abstract

Triblock copolymers such as styrene-b-(ethylene-co-butylene)-b-styrene (SEBS) have been widely used as an anion exchange membrane for fuel cells due to their phase separation properties. However, modifying the polymer architecture for optimized membrane properties is still challenging. This research develops a strategy to control the membrane morphology based on quaternized SEBS (SEBS-Q) by dual-tapering the interfacial block sequences. The structural and transport properties of SEBS-Q with various tapering styles at different hydration levels are systematically investigated by coarse-grained molecular simulations. The results show that the introduction of the tapered regions induces the formation of a bicontinuous water domain and promotes the diffusivity of the mobile components. The interplay between the solvation of the quaternary groups and the tapered fraction determines the conformation of polymer chains among the hydrophobic–hydrophilic subdomains. The strategy presented here provides a new path to fabricating fuel cell membranes with controlled microstructures.

## 1. Introduction

The anion exchange membrane fuel cell (AEMFC) is one of the zero-emission technologies for energy conversion and storage [1,2,3,4]. The high-pH operating conditions of the AEMFC make it possible to use non-precious-metal electrocatalysts, which have drawn considerable research attention in the past two decades. Significant improvements have been made in the cell performance, where the ion conductivity of the state-of-the-art AEMFC has reached the same level as the Nafion-based FC [5,6,7]. However, the peak performance relies on the loading of a platinum group metal, making full commercialization an elusive task [8]. Another long-lasting issue is the degradation of the AEM due to the attack of hydroxide ions on the polymer backbone and functional group, where most literature reports the membrane stability to be under 300 h. As a result, new synthetic strategies are still desired to improve the conductivity and durability of the AEM [9].

The performance of AEM is highly related to the polymer structure, and the block copolymer architecture is one of the most beneficial choices [10]. The immiscibility of adjacent blocks drives the polymer membrane into an intensive phase separation, and such morphology results in a high ion conductivity compared to the cases of random or homo-polymers. Many recent studies [11,12,13,14,15,16,17,18,19,20,21,22,23,24,25,26,27,28,29,30] focus on AEMs made of styrene-b-(ethylene-co-butylene)-b-styrene (SEBS) due to its high thermal and chemical stability and tunable membrane properties. The all-carbon-based polymer backbone of SEBS enhances alkaline stability compared to polymers that contain ether bonds and heteroatoms.

Various synthetic advances are made to modify SEBS-Q with the most commonly used trimethyl ammonium functional group (Q for quaternary). Mohanty et al. used C-H borylation and the Suzuki coupling reaction to elevate the degree of functionalization (DF) up to 90% per PS monomer, which overcomes the gelation issue during the chloromethylation in AEM synthesis. Nevertheless, precious transition metal catalysts are still required [11]. Earlier improvements in SEBS-Q focus on the tethered functionalized side chains, including adding ethylene-glycol side chains [13], alkyl extenders [30], alkyl spacers [14], and both alkyl side chains and alkyl spacers [21]. Recent synthetic studies focus on crosslinking the branches. Literature suggests that crosslinking quaternary groups on different polymer molecules using a short alkyl chain (Cn, n = 2–6) may alter the membrane morphology to form bicontinuous ionic cluster channels, which benefit ion transport [12]. Other studies also highlight the importance of crosslinking on cell performances [17,18,22,23,28,31] and extend the design to multi-cationic crosslinked side chains [24]. Nevertheless, the role of the side chains would require more fundamental studies to explore the relation between membrane performance [19,32] and degradation [33].

To broaden the synthetic strategy, this work adapts the tapered method to control the microstructure of the AEM by modifying the polymer backbone. Unlike the composition profile of the monomer of a regular block copolymer that changes sharply at the block junctions, a tapered block copolymer introduces a transition region that contains monomers from nearby polymer blocks. Based on the sequencing of these tapered regions, the block copolymer can be categorized as tapered or inverse-tapered. Hodrokoukes et al. have shown that the morphology of PS(polystyrene)-PI(polyisoprene) diblock copolymer can be effectively controlled by tapering the polymer sequence [34]. Kuan et al. synthesized tapered and inverse-tapered triblock polymer electrolytes and explored how the membrane morphology and ion conductivity are impacted by the taper profile and taper volume [35,36]. Considering the potential application of salt-doped block copolymer electrolytes in lithium batteries, many research works have been dedicated to exploring the mechanical and transport properties of the tapered block copolymers [37,38,39,40,41,42]. Computational investigations, mostly based on field theory and coarse-grained models, are used to obtain fundamental understandings [43,44,45,46,47]. However, most studies focus on the applications in linear block copolymers. Up to now, there has been no synthesized AEM for fuel cell applications based on tapered block copolymers.

The tapered design can significantly impact both the structure and transport properties of the hydrated AEM. In particular, changes in microphase separation, as well as the size, connectivity, and orientation of hydrophilic domains, are closely linked to ion conductivity. To investigate the membrane morphologies of tapered copolymers, mesoscale simulation methods such as dissipative particle dynamics (DPDs) are essential. In contrast to atomistic and most coarse-grained polymer models, which typically use Lennard–Jones potentials and are limited by small timesteps, DPD models polymers and solvents as soft beads with short-ranged, soft, nonbonded interactions. This allows for larger timesteps (tens of picoseconds compared to femtoseconds in atomistic models) and enhances simulation efficiency. Although DPD has some limitations [48]—such as unphysical thermodynamic properties, the loss of explicit constraints between polymer chains, and the lack of chemical detail—it remains a popular method for modeling AEMs due to its computational efficiency. DPD has been successfully applied to study the nanostructure of SEBS-Q polymers, including those with side-chain modifications [15,18,19,25,27].

This study explores the membrane performances of tapered SEBS-Q for potential use in fuel cell applications. To date, AEM based on tapered block copolymers have not been synthesized for such purposes. The paper is organized as follows: Section 2 introduces the design of AEMs based on SEBS-Q using various tapering methods, along with their coarse-grained models and the DPD force field utilized. In Section 3, the reference system composed of non-tapered SEBS-Q is validated against the work by Sepehr [15]. This section also presents the simulated morphologies of hydrated SEBS-Q with different tapered designs at various hydration levels, along with a quantitative analysis of the structure, transport properties, and polymer conformation. Finally, Section 4 summarizes the key findings from this study.

## 2. Methods

**Figure 1:**
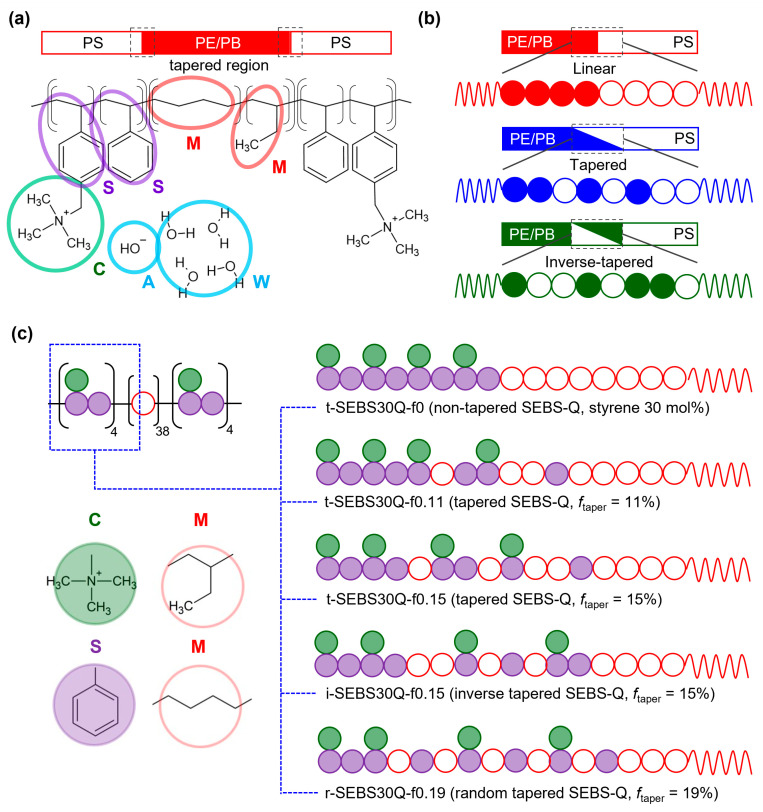
(**a**) Molecular structure and the coarse-grained representation of SEBS-Q triblock copolymer. (**b**) Monomer sequence in the tapered region (**c**) Nomenclature for the tapered SEBS30-Q models with different tapered styles (t: tapered, i: inverse-tapered, and r: random tapered) and tapered fraction *f*_taper_.

To investigate the influence of tapering, this work used SEBS-Q with no side chain modification, as shown in Figure 1a. The molar ratio of the styrene block is 30%, as used in most experimental work [11,16,18] based on the products manufactured by Kraton^TM^ Polymers. The design of the tapered block copolymers follows the schematics of the earlier works published by the Epps group [35,36,38]. The tapered region is located at both boundaries of PS-PE/PB blocks, which is modeled by altering the bead sequences. As shown in Figure 1b, the bead sequence on the block junctions is linear in non-tapered SEBS30-Q, and the modified style can be tapered or inverse-tapered based on the gradient of the bead arrangement. A random taper style was also studied based on simulation work by Seo et al. [46]. The definition of the tapered region, *f*_taper_, is the number of beads in the non-linear sequence versus the total beads in a polymer backbone. As shown in Figure 1c, the tapered region is systematically changed from 11% to 19%. The DF of the styrene block is 50%, as in reference [15], resulting in an ion exchange capacity (IEC) equivalent to 1.83 mmol/g. The hydration level *λ*, which is the number of water molecules per cationic group, varies from 4, to 8, to 16. This range of the hydration levels covers the commonly investigated range, and the corresponding water uptake (WU) equals 13%, 26%, and 53%, while 52% is the equilibrium WU for SEBS-Q with a slightly lower IEC [16].

The molecular weight M_n_ of commercial SEBS is around 118,000 g/mol, which is extremely computationally expensive to model even with coarse-grained methods such as DPD. Sepehr et al. constructed an SEBS-Q model with M_n_ of 11,840 g/mol, but the equilibrium membrane structure required 70 million steps to reach even without calculating long-ranged electrostatic interactions [15]. However, the charge-related association between anions and cationic groups plays an essential role in the structural and dynamic properties of AEM and should be taken into account. Due to the time-consuming long-ranged calculations, one needs to opt for an even shorter polymer model for a systematic study [25,27]. Nevertheless, the shortened styrene block could not provide enough mismatch interactions with the rubbery midblocks to induce the membrane structure consistent with the experimental observations.

To solve this dilemma, this work utilizes the previously developed DPD model for SEBS-Q and its force field [25,27]. The theoretical background can be found in reference [49]. The governing equations of conventional DPD simulation are concluded in Equation (1). The superscripts describe the types of pairwise forces: C for conservative forces, D for drag forces, R for random forces, and B for bonded forces. Drag and random forces control the simulation thermostat. Conservative forces describe the mismatch between different bead types via short-ranged linear force type in Equation (2). The mismatch is mainly determined by the repulsion parameter *a*_ij_ and the bead–bead distance r_ij_ related to the bead diameter r_c_. Bonded forces prevent excessive stretching and model the connectivity and rigidity of polymer chains based on the harmonic potential in Equation (3), where *K* is the bond stiffness, and r_0_ is the equilibrium bond length.

(1) $$F_{\mathrm{ij}}\left(r_{\mathrm{ij}}\right)=F_{\mathrm{ij}}^{C}+F_{\mathrm{ij}}^{D}+F_{\mathrm{ij}}^{R}+F_{\mathrm{ij}}^{B}$$

(2) $$F_{\mathrm{ij}}^{C}\left(r_{\mathrm{ij}}\right)=\left\{\begin{array}{c} a_{\mathrm{ij}}\left(1-\frac{r_{\mathrm{ij}}}{r_{c}}\right)\frac{r_{\mathrm{ij}}}{r_{\mathrm{ij}}},r_{\mathrm{ij}}<r_{c}\\ 0,r_{\mathrm{ij}}\geq r_{c} \end{array}\right.$$

(3) $$U_{\mathrm{ij}}\left(r_{\mathrm{ij}}\right)=\frac{K}{2}{\left(r_{\mathrm{ij}}-r_{0}\right)}^{2}$$

Compared to the force field constructed in the earlier works [25,27], several adjustments are made:

(1) The previous work [27] followed the framework by Sepehr et al. [15], which modeled two styrene monomers as the combination of one M (M for midblock) bead with two S (S for styrene block) beads. The revised version now models each styrene monomer as an S bead, which gives a more straightforward atom-to-bead mapping, making it easier to develop the force field parameters. The same choice is made by the Muller–Plathe group in modeling star-shaped polystyrene melts [50].
(2) The previous work [25,27] used an iterative Boltzmann inversion (IBI)-style fitting method to obtain intramolecular force field parameters. A generic rule in an earlier work [45] is adopted where the bond stiffness and length are determined based on the coarse-grained size. On this basis, the harmonic bond stiffness *K*_12_ = 80 and *K*_13_ = 40, and the equilibrium bond length *r*_12_ = 0.8 and *r*_13_ = 1.6. The purpose of this rolling back is to develop a universal force field and explicitly monitor the effects of the tapered method.
(3) This work slightly increases the molecular weight to 4378.4 g/mol of previous polymer models [27] to better explore the effects of the tapered method on membrane structures.
(4) The repulsion parameter between S (polymer end blocks) and M (polymer midblock) is tuned up to 50, corresponding to the mismatch parameter of 25. This adjustment aims to reproduce the morphology of hydrated SEBS-Q reported in the literature. It should be noted that the mismatch between A and B for ABA triblock copolymers is usually chosen to c.a. 25 to reach the desired morphology [51,52]. More details are described in the beginning of Section 3.1

The main force field parameters are summarized in Table 1. The choice of repulsion parameters *a*_ij_ reflects the thermodynamic properties, referred to as a top-down approach to obtaining coarse-grained parameters. The mismatch parameter of a bead pair i and j is defined as the difference of the repulsion parameter of bead pair i–j compared to that of pair i–i, where Δ*a*_ij_ = *a*_ij_ − *a*_ii_. To determine the mismatch between water and the polymer blocks, a calibration curve is first built by probing a solvent bead in the melted polymer. Based on the correlation $ln\left(\gamma_{i}^{\infty }\right)=0.26{∆a}_{ij}+1.35$, the experimental solubilities of water in hexadecane [53] and styrene [54] are used to estimate the infinite dilution activity coefficient and then determine the mismatch parameters between M–W and S–W pairs. Because the cationic TMA group is water-soluble, its mismatch with W is set to zero, indicating the affinity of the like bead pairs. Traditional DPD assigned reduced mass as unity for all beads, and the friction coefficient for the drag forces in Equation (2) equals 4.5 for all bead pairs [55]. It should be noted that several parameters need to be adjusted for the bead type A to model the kinetics of the hydroxide ion as illustrated in Figure 1. The reduced bead mass is 0.24 for bead A, the drag coefficient is 0.5 for the WA pair, the mismatch parameter of WA pair is −8.3, with the shorter cutoff at 0.6 *r*_c_ (1 *r*_c_ for other pairwise forces as in Equation (1), where *r*_c_ is the bead diameter). All other simulation settings, including the calculations for charged beads, are adapted from our previous work [27].

The software DL_MESO version 2.7 revision 10 [56] is used for all DPD simulations. A random configuration is created by DL_MESO based on the composition of polymeric systems. For each hydration level, the number of water, anion beads, and the IEC of the polymer are identical, so the equilibrium structure can only be affected by the style of tapering and the tapered region. Each simulation runs 1 million steps at the timestep size of 0.01 *τ* for equilibrium, followed by the production run for another 1.5 million steps. The equilibrium length of the simulation is determined by monitoring the morphological changes of the reference system (non-tapered SEBS-Q at low hydration) during the simulations. The isothermal–isobaric ensemble with the standard velocity *Verlet* integration is performed based on the Langevin barostat; the relaxation time is 0.5, and the viscosity parameter is 10, where the combined parameters yield a reasonable pressure control based on our previous experiences. The equilibrium pressure is set to 23.7, which maps to 1 atm of a standard DPD fluid. The smooth particle mesh Ewald (SPME) method is used for handling charge interactions, where the real-space convergence parameter is 0.9695, the k-value is 30 (whole box size), and the maximum b-spline order is 8. The permittivity constant is 12.6, derived based on Groot and Rabone’s equations [57]. The point charges are smeared based on the Slater-type (exponential) function [58], with the smearing parameter equal to 4.

## 3. Results and Discussion

### 3.1. The Effects of Hydration and Tapering on Membrane Structure and Properties

Based on experimental observations, a hydrated SEBS-Q is featured with nanosegregation between the hydrophilic and hydrophobic domains [16]. The hydrophilic domain is constructed by water, anions, and solvated TMA groups forming ionic clusters. For a fully hydrated SEBS-Q, the size of the ionic clusters varies from c.a. 50 nm to 100 nm based on AFM images [16] or c.a. 30 nm derived from the SAXS profile [18]. A *μ*m-level simulation is required to capture microstructures, which is extremely expensive even with coarse-grained methods. To explore the influences of tapering on the SEBS-Q, this work first reproduces a specific morphology in the literature as a reference system.

**Figure 2:**
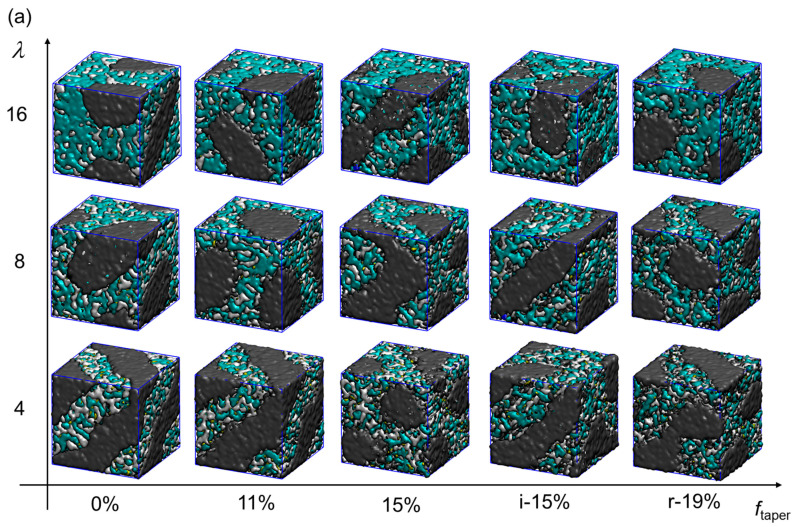

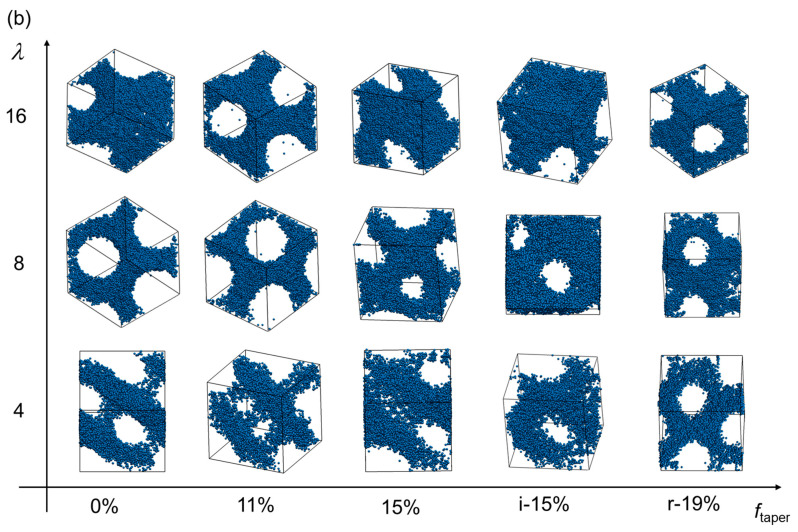
Nanostructures of the hydrated membrane at different hydration levels *λ* and tapered fractions *f*_taper_. The prefix i is for inverse taper, and r is for random taper. (a) Visualization in continuous surfaces, where PE/PB block is drawn in gray, PS in white, cationic Q groups in yellow, and water with hydroxide ion in cyan. (b) Visualization in beads representation with water and hydroxide ion only. The view angle is chosen to best represent the ordered structures.

As shown in the bottom-left panel of Figure 2a, phase separation is observed in a non-tapered (*f*_taper_ = 0%) t-SEBS30Q-f0 at low hydration (*λ* = 4). The PE/PB midblock forms a lamellae structure as a hydrophobic domain, where the functionalized PS end blocks and water form the hydrophilic domain. By visualizing the water domain, as observed in Figure 2b, the lamellar layers of the hydrophilic parts are perforated and interconnected. Judging from the continuous surface plot in Figure 2a, both water and PS form local aggregates and reside in the same domain. These observations are consistent with the findings of a similar system by Sepehr et al. [15]. Even though the morphology is reproduced by tuning the mismatch parameter between PS and PE/PB, it can be regarded as a legitimate act for a shorter polymer model. Since the compatibility of polymers is determined by the product of the Flory–Huggins parameter (χ) and the chain length (N), the shorter the polymer model, the higher the mismatch parameter required to reach the same compatibility [59].

Increasing the hydration level significantly alters the morphology of t-SEBS30Q-f0. As shown in Figure 2, the solvation of cationic TMA groups triggers a transformation in the self-assembled pattern of the ionomers. At higher hydration levels, water coalesces into a continuous phase, expanding the hydrophilic domains associated with the PS end blocks. This leads to a morphological shift in the hydrophobic domains, which evolve from spherical to cylindrical-like shapes. Simultaneously, the ionic clusters undergo a transition from an interconnected lamellar structure to a disordered, bicontinuous network. These simulated hydration effects are in good agreement with previously reported findings in the literature [15].

Meanwhile, the evolutions of the membrane morphology of SEBS30-Q with different tapered styles and regions at three different hydration levels are illustrated from left to right in Figure 2. At *λ* = 4, the lamellar structure of t-SEBS30Q-f0.11 is slightly distorted compared to that of t-SEBS30Q-f0. The water channels in Figure 2b indicate the formation of more interconnected areas between the two lamellar layers, showing the increasing compatibility of the polymer end blocks and midblock. When further increasing *f*_taper_ to 15%, two lamellar hydrophilic layers merge into a disordered structure. Nevertheless, a bicontinuous hydrophilic network is formed by r-SEBS30Q-f0.19, similar to those formed by t-SEBS30Q-f0 at *λ* = 8. On the other hand, the hydrophobic domains formed by the midblock shift from lamellar to cylinder-like structures with increasing *f*_taper_. The observation is consistent with the phase behavior of the tapered ABA triblock copolymer [34], where tapering increases the miscibility between the midblock and the end blocks, shifting the phase from being more lamellar-like to more gyroidal-like [41].

At *λ* = 8, the systems with *f*_taper_ = 0% and *f*_taper_ = 11% yield similar nanosegregations and water networks, where the bicontinuous ionic pathways are formed with some visible bottlenecks. The hydrophilic domain of t-SEBS30Q-f0.11 slightly expands, and the hydrophobic aggregates shrink compared to that of t-SEBS30Q-f0. When the tapered region increases to *f*_taper_ = 15%, the hydrophobic aggregates align in lamellar-like order. The morphologies of the hydrophilic parts for t-SEBS30Q-f0.15 and i-SEBS30Q-f0.15 are similar to the perforated lamellae observed in t-SEBS30Q-f0 at *λ* = 4. This result suggests that the increased block compatibility due to tapering has shifted the domain boundary of SEBS30-Q. Furthermore, the hydrophilic domain of i-SEBS30Q-f0.15 is swollen, indicating that inverse tapering provides a higher block compatibility than regular tapering, which is consistent with the literature observations [34].

At *λ* = 16, the hydrophilic domain is fully developed, and the hydrophobic part of t-SEBS30Q-f0 is self-assembled into long and thick aggregates. Similar to the low hydration cases, the PS end blocks and water are dispersed as two subphases within the same domain. However, the connectivity for both subphases is enhanced. For t-SEBS30Q-f0.11, the midblocks are aligned in the hexagonally packed cylinders (HEX) (indicated by the “holes” in the water network in Figure 2b), which recovers the membrane morphology of the pure SEBS30 [60]. As observed in the simulation by Sepehr et al., the HEX morphology of SEBS30 at *λ* = 0 transformed into the perforated lamellae of SEBS30-Q at *λ* = 4–8 due to the solvation of the cationic side chain. However, this influence of solvation is somehow balanced by the increased block compatibility due to tapering. This phenomenon implies that tapering can be introduced to fine-tune the membrane structure of a hydrated AEM. At a higher *f*_taper_ = 15%, the solubility of the end blocks and the midblock increases, and the domain boundaries are less well-defined. The more closely packed hydrophobic cores are visually shrunk. For r-SEBS30Q-f0.19, the “HEX”-like alignment of the midblock assembly is observed again. However, the outer edges of the hydrophobic cylinders are dissolved in the surrounding hydrophilic domain.

### 3.2. Tapering Effects on the Nanosegregation of Ionic Channels and Polymers

To evaluate the ion conductivities in the above membrane structures, the utilities built in M.Dyna*Mix* [61] is used to calculate the mean square displacement (MSD) of water (W beads) and hydroxide ions (A beads). As reported in Table 2, the diffusivity of W and A can be estimated using the Einstein relation from MSD−time plots, where $D_{A}=\langle {\left[R_{i}\left(t_{0}+t\right)-R_{i}\left(t_{0}\right)\right]}^{2}\rangle /6t$. The ion conductivities are estimated by the Nernst–Einstein equation, $\sigma =D_{A}c_{A}F^{2}/RT$, where *c*_A_ is the concentration of the A beads, and *F* is the Faraday constant (*F*^2^/*RT* = 3.7554 × 10^6^ s·S/mol at room temperature). *D*_A_ is the diffusivity of the hydroxide ions, whose physical unit is obtained by comparing the MSD with the bulk W beads (D_W_bulk_). Although it is a common practice to calculate the membrane conductivity of fuel cell membranes in simulations [62,63], it should be noted that the Nernst–Einstein approximation is strictly valid for the dilute ion condition. For our reference system, the ion conductivity roughly agrees with the available experimental measurement [16]. As reported in Table 2, the non-tapered SEBS30-Q (IEC = 1.83) has a conductivity of 45.7 mS/cm at *λ* = 16 (53% WU), compared to 35 mS/cm for SEBS30-Q (IEC = 1.35) at 52% WU [16].

As observed in Figure 2, the water domain develops with the hydration level, providing a more efficient pathway for water transportation, and the diffusion coefficient of the water monotonically increases with *λ*, as reported in Table 2. However, water mobility slightly decreases with the tapered fraction at all hydration levels. On the contrary, the ratio of *D*_A_ and *D*_W_ increases with the tapered fraction. As a result, the tapered SEBS30-Q provides ion conductivities similar to those of the non-tapered polymers, based on diverse membrane morphologies. To rationalize these observations, structural analyses, including the pore size distribution (PSD) and the radial distribution function (RDF), are performed. The RDF is calculated using the GPU-accelerated codes in the VMD software (version 1.9.4a53) [64]. It should be noted that the unit of length in RDF and PSD is a reduced value normalized by bead diameter (*r*_c_). The decision not to map length to the physical unit using literature correlation [57] is based on the reasoning of a reference [15]. There existed a scaling difference between the simulation morphology and the scattering experimental, mainly due to the small molecular weight of the polymer model [15].

**Figure 3:**
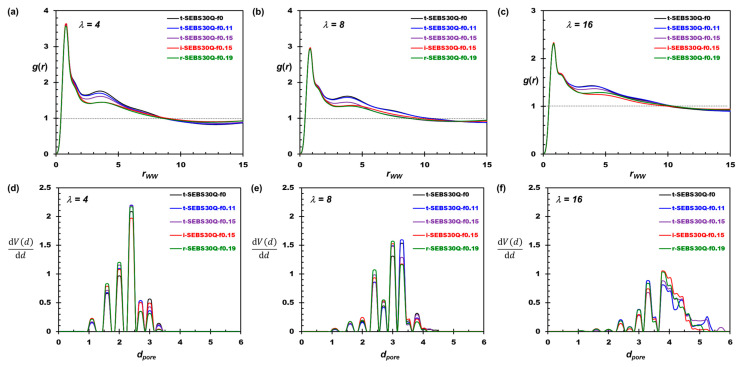
Structural characteristics of the water channels. (**a**–**c**) The radial distribution function (RDF) of WW bead pairs. (**d**–**f**) The pore size distribution (PSD) of the water domain.

As shown in Figure 3a–c, the correlation of WW bead pairs reveals the percolation of water channels. The position of *g*(*r*_WW_) crossing unity (starts decorrelating) shifts to larger distances, which quantifies the growth of the hydrophilic domains observed in Section 3.1. The PSD is calculated using PoreBlazer codes on the equilibrium morphology [65]. The hydrated membrane structure is first digitized into a lattice model using the in-house program, where each lattice site is considered a “void” if it overlaps with any W bead. The geometry of these void domains is then probed by a test particle based on Monte Carlo movements. The distribution of all possible geometries for water aggregates is illustrated in Figure 3d–f. If the water aggregates form a continuous channel across at least one dimension of the simulation box, the maximum (*d*_max_) and the minimum (*d*_min_) channel diameters are calculated as reported in Table 2.

As shown in Figure 3d, the peaks of PSD at small *d_pore_* values indicate the local and dispersed water aggregates in the hydrophilic domain co-constructed with polymer end blocks at *λ* = 4. At a higher *λ*, the PSD profiles in Figure 3e,f shift toward larger pore diameters showing the expansion of the hydrophilic domain, and the continuous water domain forms at *λ* = 16. The PSD profiles of tapered copolymers are similar to the non-tapered ones, and the geometry of the bottleneck (*d*_min_) reported in Table 2 is unaffected by the tapered fraction. This indicates the local aggregation behavior of water is mainly determined by the solvation of the cationic groups while tapering affects the agglomeration of water aggregates. As the tapered region is introduced to increase block compatibility, the boundary between hydrophilic and hydrophobic domains becomes indistinct, thus increasing the “roughness” of the domain boundaries. The maximum pore size (*d*_max_) reported in Table 2 is shrunk as *f*_taper_ rises, and the PSD profiles shift toward the medium pore sizes, as shown in Figure 3. Nevertheless, two exceptions are observed. For t-SEBS30Q-f0.11 at *λ* = 16, the HEX morphology is formed for the hydrophobic domain, and, for t-SEBS30Q-f0.15 at *λ* = 8, the layered arrangement is observed for the midblock assembly. The polymeric cores packed in an orderly manner enhance the efficiency of water transport in the water channel, as indicated by the substantially higher *d*_max_ and the diffusion coefficient of water in Table 2.

**Figure 4:**
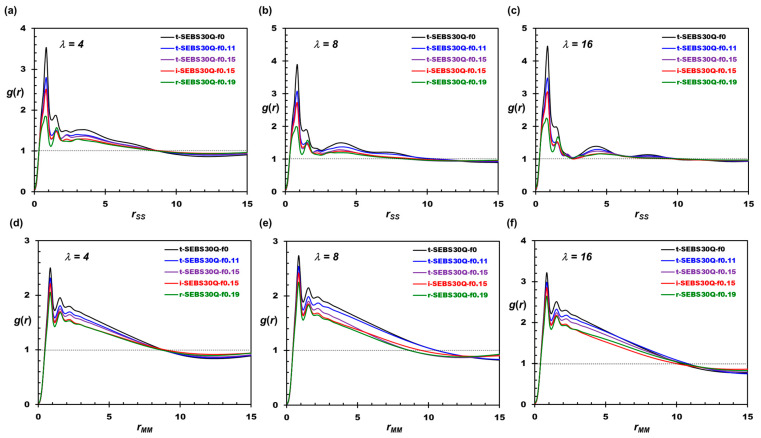
Structural characteristics of the tapered SEBS30-Q polymer. (**a**–**c**) The radial distribution function (RDF) of SS bead pairs. (**d**–**f**) The radial distribution function (RDF) of MM bead pairs.

Figure 4 illustrates the RDF distribution of the tapered SEBS30-Q polymers, where the RDF of SS bead pairs shows the correlation for polymer end blocks, and the RDF of MM bead pairs dictates the correlation for the polymer midblock. Similar to the profile for WW, the expansion of the hydrophilic domain can be seen in Figure 4a–c, where the position of *g*(*r*_SS_) crossing unity shifts to a larger separation as the hydration level increases. Moreover, the height of the first peak is elevated as *λ* rises. This behavior suggests that the hydrophobic cores are arranged in a more orderly manner when the water content increases, consistent with the literature observations [15]. Although the SS RDF profiles resemble the WW ones, *g*(*r*_SS_) shows weaker correlations than *g*(*r_WW_*) after the first peak, indicating the stronger affinity of water to form a continuous phase in the hydrophilic domain. As *f*_taper_ increases, the height of the first peak decreases in Figure 4a–c, suggesting the increased compatibility between the PS and PE blocks. The inverse-tapered system provides improved block compatibility compared to regular-tapered ones with the same *f*_taper_, as evidenced by the reduced correlations after the first peak. The undermined phase separation is also observed in the correlation of the midblock. The first peak of *g*(*r*_MM_) in Figure 4d–f grows with water uptake but decreases with the tapered fraction. It should be noted that t-SEBS30Q-f0.11 develops stronger long-ranged correlations for *r*_MM_ greater than 8R_c_ compared to t-SEBS30Q-f0 as *λ* increases, which indicates the formation of HEX.

**Figure 5:**
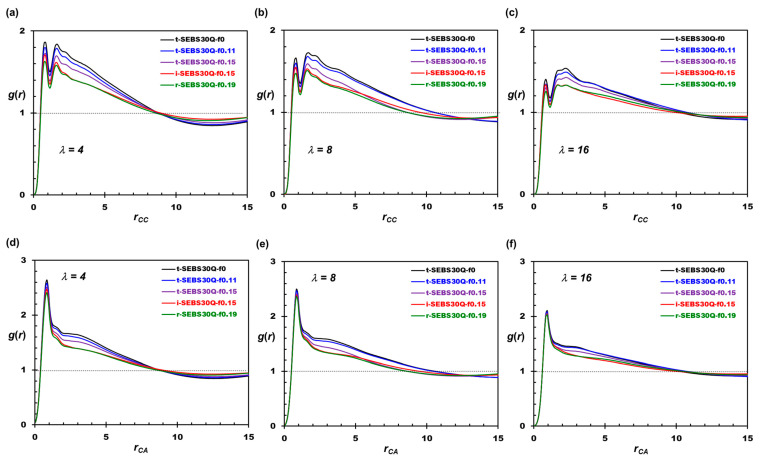
Association between the cationic group C and anion A. (**a**–**c**) The radial distribution function (RDF) for CC bead pairs. (**d**–**f**) RDF for CA bead pairs.

Based on the ab initio MD simulations [66], the movement of hydroxide ions is related to their networking with water and surrounding cationic groups. The association between water and hydroxide is predicted by the DPD hydroxide model developed in the author’s previous works [25,27]. The transportation of hydroxide ions is carried out by mixed structural and vehicular mechanisms, which are described implicitly by the developed model. The association between cationic groups and anions is modeled by the electrostatic interactions. A well-structured water network enhances ion conductivity by promoting the vehicular diffusion of hydroxide ions. However, the alignment of the cationic groups plays a substantial role in the structural diffusion of hydroxide ions. In our schematics of tapering, the locations of the two cationic groups closest to the polymer ends are fixed. The other cationic groups shift their tethered position to the middle of the polymer as *f*_taper_ is increased. In Figure 5a–c, the decreased first peak with higher hydration shows that the cationic groups are more solvated in water and hydrophilic channels are expanded. At *λ* = 4, the CC pairs have a weakened correlation as the tapered region is enlarged, which increases the average distance between the two cationic groups. This effect decreases the probability of hydroxide ions being associated with two cationic groups when traveling through the confined space between the two groups. The enhanced dissociation between cationic groups and anions can also be observed in Figure 5d–f, where the correlations in *g*(*r*_CA_) become weaker with increased *f*_taper_. As reported in Table 2, the diffusion coefficient of hydroxide ions is generally increased with *f*_taper_, and the lower *D*_A_ for the inverse-tapered form is caused by the hindered water diffusion. At *λ* = 8, the CA bead pairs decorate the fastest for t-SEBS30Q-f0.15 and r-SEBS30Q-f0.19, corresponding to the highest D_A_/D_W_ ratios in Table 2. At *λ* = 16, t-SEBS30Q-f0.11 has the highest D_A_/D_W_ and ion conductivity. It is hence concluded that the fraction of the tapered region should be balanced with the water content to reach the best ion conductivity. A high *f*_taper_ may alter the phase transition of the SEBS-Q membrane at high hydration levels, where the local aggregates of the hydrophobic midblock dissolve in the hydrophilic domains and disturb the ion transportation. Because of the soft-core potential employed in DPD, the diffusivity of water and anions in the practical cases is expected to be lower than the predicted values reported in Table 2.

### 3.3. Tapering Effects on Polymer Conformations

**Figure 6:**
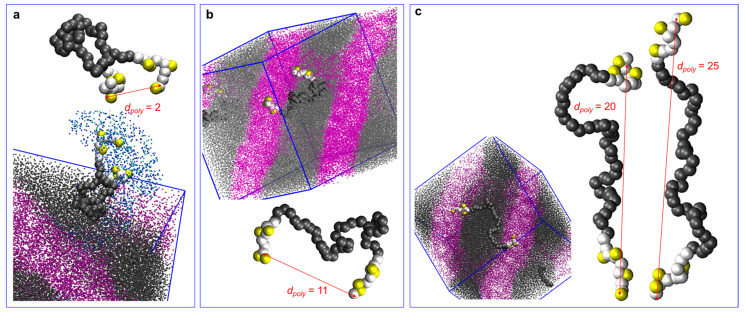
Representative polymer conformations in the nanosegregation. In the reference system, the M (gray dots) and S blocks (purple dots) form interconnected lamellae. (**a**) The folded structure has an end-to-end distance (*d_poly_*) of around 2R_c_. (**b**) The folded structure with a larger *d_poly_*. (**c**) The bridged structure with *d_poly_* larger than 20R_c_.

The nanosegregated morphology is highly related to the corresponding polymer conformations. By analyzing the end-to-end distance (*d*_poly_), calculated from the separation between the first and the last beads on the polymer backbone, the conformations can be categorized as folded (looped) or stretched (bridged) ones. The reference system is utilized to demonstrate the difference between the two conformations. As shown in Figure 6a, a chain in the looped conformation has both end blocks residing in the same subdomain. The midblock forms the rubbery aggregates, and the functionalized side chains on the end blocks associate with water and hydroxide ions, forming the ionic channels. The value of *d*_poly_ varies based on the location of the polymer. In some cases, as in Figure 6b, one of the end blocks resides in the perforated area between two layers of the polystyrene, resulting in a slightly larger *d*_poly_. As shown in Figure 6c, a chain in the bridged conformation has end blocks located in different subdomains. The conformation of the midblock effectively determines the equilibrium domain size of the rubbery PS region.

**Figure 7:**
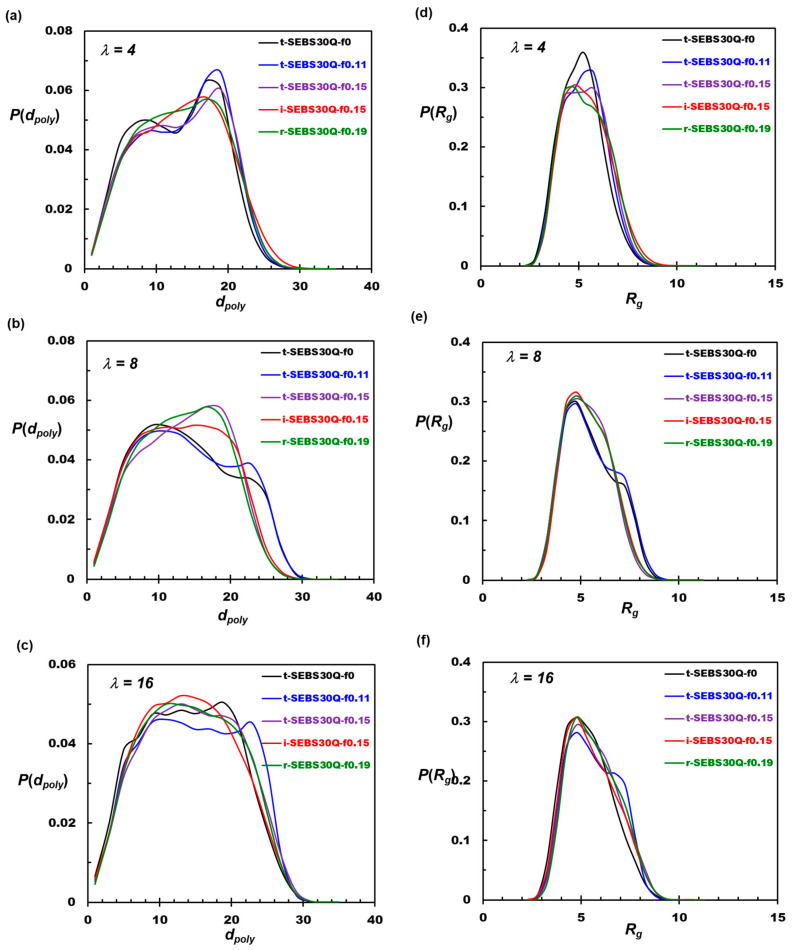
The distribution functions of (**a**–**c**) end-to-end distances and (**d**–**f**) radii of gyration of tapered SEBS-30Q at different tapered fractions and hydration levels.

Figure 7 compares the probability distributions of the polymer end-to-end distance and radius of gyration. The radius of gyration (*R_g_*) measures the distribution of atoms in the molecular structure related to the center of mass of the polymer chain. For a polymer chain with N beads, the radius of gyration *R_g_* is calculated as Equation (4):

(4) $${R_{g}}^{2}=\frac{\sum_{i=1}^{N}m_{i}{\left(r_{i}-r_{CM}\right)}^{2}}{\sum_{i=1}^{N}m_{i}}$$

where r*_i_* is the coordinate of the bead *i* in the polymer, m*_i_* is the mass of the bead *i* in the polymer, and r*_CM_* is the coordinate of the center of mass of the polymer. A large *R_g_* corresponds to atoms located away from the center of the molecule.

At *λ* = 4, two prominent peaks are observed for the *d_poly_* of t-SEBS30Q-f0 located at 8 and 18 bead diameters in Figure 7a, showing mixed conformations for looping and stretching. For t-SEBS30Q-f0.11, the growth in the second peak and the diminished first peak suggest that increased block compatibility favors the stretched conformation for the polymers. For higher *f*_taper_, the distinction between the two peaks gradually diminishes, and the probability of a higher end-to-end distance increases. Specifically, for the 15% inverse-tapered and 19% randomly tapered copolymers, Figure 7a,d show a more uniform polymer conformation.

At *λ* = 8, the first peak is more dominant with a shorter end-to-end distance and radius of gyration for t-SEBS30Q-f0 and t-SEBS30Q-f0.11, indicating that the solvation of the cationic groups favors the folded conformations as presented in Figure 7b,e. This observation suggests that fully stretched conformations are less favorable for the tapered copolymers as the water content is increased. The slight shrink in the polymer chain may be attributed to the interaction of end blocks with water. As the tapered region increases, more hydrophobic monomers relocate to the end block regions. The surrounding water is a good solvent for the functionalized cationic groups but is a bad solvent for the exposed PS and PE. This mismatched interaction favors the folded conformation of the end blocks to align more charged groups in the PS–water interphase. The observations here are consistent with the hypothesis in the literature [41]; as the miscibility increases with tapering, the tendency of phase separation decreases, and the conformational entropy also declines, which makes it easier to locate two block junctions at the domain interface.

At the fully hydrated condition *λ* = 16, the formation of a continuous water domain suppresses the phase separation of PS and PE. The radius of gyration and end-to-end profiles show similar characteristics, as seen in Figure 7c,f. It is observed that the slightly tapered t-SEBS30Q-f0.11 may develop fully stretched conformations. This behavior can be explained by the excess solvation of the functional groups at the end blocks overcoming the penalty of solvating the hydrophobic monomers between the functionalized monomers. The stretched conformation is also related to the formation of the ordered structure, as observed in the visualization illustrated in Figure 2. As discussed in Section 3.1 and Section 3.2, such an ordered structure promotes ion conduction. Moreover, the self-assembly of the bridge conformations, together with the occurrence of chain entanglement, will vitrify the hydrophobic domains and enhance the membrane’s mechanical properties. It is concluded that introducing tapered designs at *f*_taper_ = 11% to 15% will improve the overall membrane performance of the TMA-functionalized SEBS AEM in terms of ion conductivity and mechanical strength. Excessive tapering may reduce the mismatch between the hydrophobic and hydrophilic domains and suppress phase separation, especially in fully hydrated working conditions.

## 4. Conclusions

This study introduces a new design strategy for tapering functionalized SEBS-based triblock copolymers in developing AEM fuel cells. The designed polymers include tapered, inverse-tapered, and random-tapered forms featuring 11% to 19% tapered regions. The structural and transport properties of these polymer membranes were analyzed across hydration levels *λ* from 4 to 16 using mesoscale DPD simulations. The simulation model was validated by reproducing the morphologies of non-tapered SEBS-Q membranes with varying water contents, as reported in the literature [15].

The simulation results show that the hydration effects of tapered SEBS-Q polymers are similar to those of the basic non-tapered form, with water forming a continuous phase that enhances transport properties. However, tapering improves the miscibility of polymer blocks and alters the hydrated membrane structure at a fixed water content. At low hydration levels, tapering transforms the interconnected lamellar structure of the hydrophilic domain into a bicontinuous network. In contrast, at higher hydration levels, it induces a transition from disordered structures to ordered ones, such as lamellar or hexagonal (HEX). Despite this, increased miscibility can lead to rougher phase boundaries in the hydrophilic domain, which impedes water transport. Nonetheless, tapering reduces the likelihood of two cationic groups associating with a single anion, thereby enhancing ion transport. This enhancement in ion transport, despite the potential decrease in water transport efficiency due to increased miscibility, could lead to improved AEM fuel cell performance. Although excessive tapering may decrease transport efficiency, polymers with moderately tapered regions promote the formation of ordered structures at a high water content, improving hydrophilic channels and overall transport properties. The analysis of polymer conformations further supports these findings, showing that moderately tapered copolymers tend to adopt stretched conformations, leading to ordered structures and enlarged hydrophobic domain sizes.

Tapering presents a promising alternative to sidechain modification for controlling the microstructure of hydrated anion exchange membranes (AEMs) made from functionalized triblock copolymers. While sidechain modification is widely used to enhance AEM conductivity, this study explores tapering as a distinct approach. Moreover, the combination of tapering and sidechain modifications could broaden the design possibilities for future AEM development. Nevertheless, experimental validation is required to confirm these findings. The proposed polymers may be synthesized by incorporating tapered regions via anionic copolymerization [34], followed by a sequential process to produce SEBS-Q membranes [29]. Ongoing research, which will examine the effects of tapering on water uptake [67], membrane deformation, and its integration into advanced triblock copolymer-based AEM designs [68], aims to provide a comprehensive understanding of the potential of tapering in AEM development.

## Figures and Tables

**Table 1:** Force field parameters of Equations (2) and (3) for DPD simulations. The subscription of bond parameters refers to the connected bead pair, where the “12” bonds apply on the nearest neighbor for bead connectivity, and “13” bonds apply on the 2nd nearest neighbor for polymer rigidity. Unlike regular MD simulations, the pairwise forces in Equation (1) are still applied for bonded beads.

Repulsion parameters					
*a*_ij_	M	S	C	W	A
M	25.0				
S	50.0	25.0			
C	46.5	41.2	25.0		
W	46.5	41.2	25.0	25.0	
A	25.0	25.0	25.0	−8.3	25.0
**Bond parameters**					
Harmoic	*K*_(12)_	r_0(12)_	*K*_(13)_	r_0(13)_	
	80.0	0.8	40.0	1.6	

**Table 2:** Transport and structural properties of tapered SEBS30-Q block copolymers.

Polymer.	*λ*	D_W_/D_W_bulk_	D_A_/D_W_bulk_	D_A_/D_W_	*σ* [mS/cm]	d_min_(*R*_c_)	d_max_ (*R*_c_)
t-SEBS30Q-f0	4	0.27	0.50	1.85	18.7	2.1	3.3
t-SEBS30Q-f0.11	4	0.26	0.51	1.97	18.8	2.1	3.3
t-SEBS30Q-f0.15	4	0.25	0.52	2.08	19.4	2.1	3.3
i-SEBS30Q-f0.15	4	0.23	0.49	2.11	18.3	2.1	3.3
r-SEBS30Q-f0.19	4	0.23	0.51	2.22	19.0	2.1	3.3
t-SEBS30Q-f0	8	0.33	0.56	1.69	18.8	2.8	4.5
t-SEBS30Q-f0.11	8	0.34	0.56	1.64	18.8	2.8	4.1
t-SEBS30Q-f0.15	8	0.32	0.56	1.74	18.8	2.8	4.3
i-SEBS30Q-f0.15	8	0.31	0.51	1.65	17.2	2.8	4.1
r-SEBS30Q-f0.19	8	0.30	0.55	1.81	18.5	2.8	4.1
t-SEBS30Q-f0	16	0.93	1.65	1.77	45.7	3.6	5.4
t-SEBS30Q-f0.11	16	0.84	1.70	2.02	47.3	3.6	5.7
t-SEBS30Q-f0.15	16	0.90	1.59	1.78	44.3	3.6	5.4
i-SEBS30Q-f0.15	16	0.99	1.67	1.68	46.3	3.6	5.2
r-SEBS30Q-f0.19	16	0.84	1.50	1.78	41.8	3.6	5.1

## Data Availability

The simulation data supporting the findings of this study are available from the corresponding author upon reasonable request.

## References

[1] Park E.J., Jannaschet P., Miyatake K., Bae C., Noonan K., Fujimoto C., Holdcroft S., Varcoe J.R., Henkensmeier D., Guiver M.D. (2024). Aryl ether-free polymer electrolytes for electrochemical and energy devices. Chem. Soc. Rev..

[2] Song W., Zhang X., Yang C., Yang Z., Wu L., Ge X.L., Xu T. (2023). Alkaline Membranes toward Electrochemical Energy Devices: Recent Development and Future Perspectives. ACS Cent. Sci..

[3] Chen N.J., Lee Y.M. (2022). Anion-conducting polyelectrolytes for energy devices. Trends Chem..

[4] Mustain W.E., Chatenet M., Page M., Kim S.Y. (2020). Durability challenges of anion exchange membrane fuel cells. Energy Environ. Sci..

[5] Varcoe J.R., Atanassov P., Dekel D.R., Herring A.M., Hickner A.M., Kohl A.P., Kucernak R.A., Mustain E.W., Nijmeijer K., Scott K. (2014). Anion-exchange membranes in electrochemical energy systems. Energy Environ. Sci..

[6] Dekel D.R. (2018). Review of cell performance in anion exchange membrane fuel cells. J. Power Sources.

[7] Gottesfeld S., Dekel R.D., Page M., Bae C., Yan Y., Zelenay P., Kim Y.S. (2018). Anion exchange membrane fuel cells: Current status and remaining challenges. J. Power Sources.

[8] Li F., Chan S.H., Tu Z. (2024). Recent Development of Anion Exchange Membrane Fuel Cells and Performance Optimization Strategies: A Review. Chem. Rec..

[9] You W., Noonan K.J.T., Coates G.W. (2020). Alkaline-stable anion exchange membranes: A review of synthetic approaches. Prog. Polym. Sci..

[10] Elabd Y.A. (2019). Ion transport in hydroxide conducting block copolymers. Mol. Syst. Des. Eng..

[11] Mohanty A.D., Ryu C.Y., Kim S.Y., Bae C. (2015). Stable Elastomeric Anion Exchange Membranes Based on Quaternary Ammonium-Tethered Polystyrene-b-poly(ethylene-co-butylene)-b-polystyrene Triblock Copolymers. Macromolecules.

[12] Dai P., Mo Z.H., Xu R.W., Zhang S., Wu Y.X. (2016). Cross-Linked Quaternized Poly(styrene-b-(ethylene-co-butylene)-b-styrene) for Anion Exchange Membrane: Synthesis, Characterization and Properties. ACS Appl. Mater. Interfaces.

[13] Yang C., Wang S., Ma W., Zhao S., Xu Z., Sun G. (2016). Highly stable poly(ethylene glycol)-grafted alkaline anion exchange membranes. J. Mater. Chem. A.

[14] Lin C.X., Wang X.Q., Hu E.N., Yang Q., Zhang Q.G., Zhu A.M., Liu Q.M. (2017). Quaternized triblock polymer anion exchange membranes with enhanced alkaline stability. J. Membr. Sci..

[15] Sepehr F., Liu H., Luo X., Bae C., Tuckerman M.E., Hickner M.A., Paddison S.J. (2017). Mesoscale Simulations of Anion Exchange Membranes Based on Quaternary Ammonium Tethered Triblock Copolymers. Macromolecules.

[16] Wang Z.Y., Parrondo J., Ramani V. (2017). Anion Exchange Membranes Based on Polystyrene-Block-Poly(ethylene-ran-butylene)-Block-Polystyrene Triblock Copolymers: Cation Stability and Fuel Cell Performance. J. Electrochem. Soc..

[17] Hao J.K., Gao X., Jiang Y., Zhang H., Luo J., Shao Z., Yi B. (2018). Crosslinked high-performance anion exchange membranes based on poly (styrene-b-(ethylene-co-butylene)-b-styrene). J. Membr. Sci..

[18] Jeon J.Y., Park S., Han J., Maurya S., Mohanty A.D., Tian D., Saikia N., Hickner M.A., Ryu C.Y., Tuckerman M.E. (2019). Synthesis of Aromatic Anion Exchange Membranes by Friedel-Crafts Bromoalkylation and Cross-Linking of Polystyrene Block Copolymers. Macromolecules.

[19] Luo X., Paddison S.J. (2019). DPD simulations of anion exchange membrane: The effect of an alkyl spacer on the hydrated morphology. Solid State Ion..

[20] Zhu Z., Luo X., Paddison S.J. (2019). DPD simulations of anion exchange membranes functionalized with various cationic groups and associated anions. Solid State Ion..

[21] Al Munsur A., Hossain I., Nam S.Y., Chae J.E., Kim T.H. (2020). Hydrophobic-hydrophilic comb-type quaternary ammonium-functionalized SEBS copolymers for high performance anion exchange membranes. J. Membr. Sci..

[22] Al Munsur A., Hossain I., Nam S.Y., Chae J.E., Kim T.H. (2020). Quaternary ammonium-functionalized hexyl bis(quaternary ammonium)-mediated partially crosslinked SEBSs as highly conductive and stable anion exchange membranes. Int. J. Hydrogen Energy.

[23] Gao X., Yu H.M., Xie F., Hao J., Shao Z. (2020). High performance cross-linked anion exchange membrane based on aryl-ether free polymer backbones for anion exchange membrane fuel cell application. Sustain. Energy Fuels.

[24] Li Z., Li C., Long C., Sang J., Tian L., Wang F., Wang Z., Zhu H. (2020). Elastic and durable multi-cation-crosslinked anion exchange membrane based on poly(styrene-b-(ethylene-co-butylene)-b-styrene). J. Polym. Sci..

[25] Lee M.T. (2021). Designing Highly Conductive Block Copolymer-Based Anion Exchange Membranes by Mesoscale Simulations. J. Phys. Chem. B.

[26] Wang F., Li C., Sang J., Cui Y., Zhu H. (2021). Synthesis and characterization of a long side-chain double-cation crosslinked anion-exchange membrane based on poly(styrene-b-(ethylene-co-butylene)-b-styrene). Int. J. Hydrogen Energy.

[27] Chen Q.G., Lee M.T. (2022). Anion Exchange Membranes for Fuel Cells Based on Quaternized Polystyrene-b-poly(ethylene-co-butylene)-b-polystyrene Triblock Copolymers with Spacer-Sidechain Design. Polymers.

[28] Han J., Liu C., Deng C., Zhang Y., Song W., Zheng X., Liu X., Zhang Y., Yang X., Ren Z. (2022). Mechanically robust and highly conductive semi-interpenetrating network anion exchange membranes for fuel cell applications. J. Power Sources.

[29] Liu F., Wahid U., Zhao Z., Liu W., Zhang C. (2022). Design, synthesis and characterization of SEBS anion exchange membranes with ultrahigh dimensional stability. J. Polym. Res..

[30] Sang J., Yang L., Li Z., Wang F., Wang Z., Zhu H. (2022). Comb-shape d SEBS-base d anion exchange membranes with obvious microphase separation morphology. Electrochim. Acta.

[31] Wang Z., Li Z., Chen N., Lu C., Wang F., Zhu H. (2018). Crosslinked poly (2,6-dimethyl-1,4-phenylene oxide) polyelectrolyte enhanced with poly (styrene-b-(ethylene-co-butylene)-b-styrene) for anion exchange membrane applications. J. Membr. Sci..

[32] Rezayani M., Sharif F., Makki H. (2022). Understanding ion diffusion in anion exchange membranes; effects of morphology and mobility of pendant cationic groups. J. Mater. Chem. A.

[33] Erimban S., Bombau J.I., Karnes J.J., Molinero V. (2024). Degradation of Anion Exchange Membranes by Cation Elimination: Impact on Water Uptake, Nanostructure, and Ionic Mobility. J. Phys. Chem. C.

[34] Hodrokoukes P., Floudas G., Pispas S., Hadjichristidis N. (2001). Microphase Separation in Normal and Inverse Tapered Block Copolymers of Polystyrene and Polyisoprene. 1. Phase State. Macromolecules.

[35] Kuan W., Roy R., Rong L., Hsiao B.S., Epps T.H. (2012). Design and Synthesis of Network-Forming Triblock Copolymers Using Tapered Block Interfaces. ACS Macro Lett..

[36] Kuan W.F., Remy R., Mackay M.E., Epps T.H. (2015). Controlled ionic conductivity via tapered block polymer electrolytes. Rsc. Adv..

[37] McIntosh L.D., Kubo T., Lodge T.P. (2014). Morphology, Modulus, and Conductivity of a Triblock Terpolymer/Ionic Liquid Electrolyte Membrane. Macromolecules.

[38] Young W.S., Kuan W.F., Epps T.H. (2014). Block Copolymer Electrolytes for Rechargeable Lithium Batteries. J. Polym. Sci. Part B-Polym. Phys..

[39] Patterson A.L., Danielsen S.P.O., Yu B., Davidson E.C., Fredrickson G.H., Segalman R.A. (2019). Sequence Effects on Block Copolymer Self-Assembly through Tuning Chain Conformation and Segregation Strength Utilizing Sequence-Defined Polypeptoids. Macromolecules.

[40] Patterson A.L., Danielsen S.P.O., Yu B., Davidson E.C., Fredrickson G.H., Segalman R.A. (2020). Monomer Sequence Effects on Interfacial Width and Mixing in Self-Assembled Diblock Copolymers. Macromolecules.

[41] Steube M., Johann T., Barent D.R., Müller A.H.E., Frey H. (2022). Rational design of tapered multiblock copolymers for thermoplastic elastomers. Prog. Polym. Sci..

[42] Gavrilov A.A., Potemkin I.I. (2023). Copolymers with Nonblocky Sequences as Novel Materials with Finely Tuned Properties. J. Phys. Chem. B.

[43] Luo M., Brown J.R., Remy R.A., Scott D.A., Mackay M.E., Hall L.M., Epps T.H. (2016). Determination of Interfacial Mixing in Tapered Block Polymer Thin Films: Experimental and Theoretical Investigations. Macromolecules.

[44] Sethuraman V., Ganesan V. (2016). Segmental dynamics in lamellar phases of tapered copolymers. Soft Matter.

[45] Brown J.R., Seo Y., Sides S.W., Hall L.M. (2017). Unique Phase Behavior of Inverse Tapered Block Copolymers: Self Consistent Field Theory and Molecular Dynamics Simulations. Macromolecules.

[46] Seo Y., Sides S.W., Hall L.M. (2017). Diffusion of Selective Penetrants in Interfacially Modified Block Copolymers from Molecular Dynamics Simulations. Acs Macro Lett..

[47] Zhang H., Riggleman R.A. (2023). Percolation of co-continuous domains in tapered copolymer networks. Mol. Syst. Des. Eng..

[48] Español P., Warren P.B. (2017). Perspective: Dissipative particle dynamics. J. Chem. Phys..

[49] Frenkel D., Smit D. (2001). Understanding Molecular Simulation.

[50] Wu Z.H., Muller-Plathe F. (2022). Slip-Spring Hybrid Particle-Field Molecular Dynamics for Coarse-Graining Branched Polymer Melts: Polystyrene Melts as an Example. J. Chem. Theory Comput..

[51] Droghetti H., Pagonabarraga I., Carbone P., Asinari P., Marchisio M. (2018). Dissipative particle dynamics simulations of tri-block co-polymer and water: Phase diagram validation and microstructure identification. J. Chem. Phys..

[52] Lin Y.L., Chang H.Y., Sheng Y.J., Tsao H.K. (2014). Self-assembled polymersomes formed by symmetric, asymmetric and side-chain-tethered coil-rod-coil triblock copolymers. Soft Matt..

[53] Shaw D.G., Maczynski A., Goral M., Wisniewska-Goclowska B., Skrzecz A., Owczarek I., Blazej K., Haulait-Pirson M.C., Hefter G.T., Kapuku F. (2006). IUPAC-NIST solubility data series. 81. Hydrocarbons with water and seawater-revised and updated. Part 11. C-13-C-36 hydrocarbons with water. J. Phys. Chem. Ref. Data.

[54] Thomas S.V., Thomas S.V., Visakh P.M. (2011). Handbook of Engineering and Speciality Thermoplastics: Polyethers and Polyesters.

[55] Groot R.D., Warren P.B. (1997). Dissipative particle dynamics: Bridging the gap between atomistic and mesoscopic simulation. J. Chem. Phys..

[56] Seaton M.A., Anderson R.L., Metz s., Smith W. (2013). DL_MESO: Highly scalable mesoscale simulations. Mol. Simul..

[57] Groot R.D., Rabone K.L. (2001). Mesoscopic simulation of cell membrane damage, morphology change and rupture by nonionic surfactants. Biophys. J..

[58] Gonzalez-Melchor M., Mayoral E., Velázquez M.E., Alejandre J. (2006). Electrostatic interactions in dissipative particle dynamics using the Ewald sums. Biophys. J..

[59] Prochazka K., Limpouchová Z., Štěpánek M., Šindelka K., Lísal M. (2022). DPD Modelling of the Self- and Co-Assembly of Polymers and Polyelectrolytes in Aqueous Media: Impact on Polymer Science. Polymers.

[60] Heck B., Arends P., Ganter M., Kressler J., Stühn B. (1997). SAXS and TEM Studies on Poly(styrene)-block-poly(ethene-co-but-1-ene)-block-poly(styrene) in Bulk and at Various Interfaces. Macromolecules.

[61] Lyubartsev A.P., Laaksonen A.M. (2000). DynaMix–a scalable portable parallel MD simulation package for arbitrary molecular mixtures. Comput. Phys. Commun..

[62] Kreuer K.D., Paddison S.J., Spohr E., Schuster M. (2004). Transport in proton conductors for fuel-cell applications: Simulations, elementary reactions, and phenomenology. Chem. Rev..

[63] Kusoglu A., Weber A.Z. (2017). New Insights into Perfluorinated Sulfonic-Acid lonomers. Chem. Rev..

[64] Humphrey W., Dalke A., Schulten K. (1996). VMD: Visual molecular dynamics. J. Mol. Graph. Model..

[65] Sarkisov L., Bueno-Perez R., Sutharson M., Fairen-Jimenez D. (2020). Material Informatics with PoreBlazer v4.0 and CSD MOF Database. Chem. Mater..

[66] Castaneda S., Ribadeneira R. (2020). Description of Hydroxide Ion Structural Diffusion in a Quaternized SEBS Anion Exchange Membrane Using Ab Initio Molecular Dynamics. J. Phys. Chem. C.

[67] Dorenbos G. (2023). Simulated and Experimental Trends Regarding Water Uptake in Polymeric Electrolyte Membranes. J. Phys. Chem. B.

[68] Allushi A., Bakvand P.M., Gong H., Jannasch P. (2023). Hydroxide conducting BAB triblock copolymers tailored for durable high-performance anion exchange membranes. Mater. Adv..

